# 

**Published:** 1890-12-27

**Authors:** 


PRICE ONE PENNY.

No. 222, Vol. IX.—December 27th, 1890.

[Registered at the General Post Office as a Newspaper for transmission in the United Kingdom and Abroad.]

WITH EXTRA NURSING SUPPLEMENT

Per Annum, 6s. 6d.; post free.

Monthly Parts, 6d.; post free 8d.

Ditto per Annum, 8s., post free.

### THE HOSPITAL

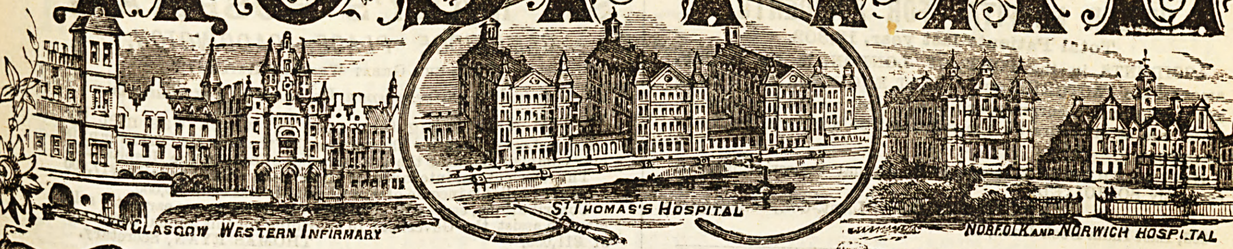

A WEEKLY JOURNAL OF

### Science, Medicine, Nursing and Philanthropy.

SATURDAY, DECEMBER 27th, 1890.

**A Sober Critic of Koch** . . . 197

**Passing Topics.**—The Season of Appeals . . . 198

**The Doctor's Armchair.**—Kosher and Trifa . . . 199

**The History and Capabilities of Herbal Simples.** By W. T. Fernie, M.D.—XXV.—Cress (*Continued*) . . . 200

**Turning Over New Leaves.**—Has Pasteur Failed? . . . 201

**Hypnotism** . . . 202

**Everybody's Page** . . . 203

**Notes and News.** . . . 204

**Annotations.**—Diphtheria at Marlborough School—Wholesome Agnosticism Wanted—Ill-Health and Crippled Powers . . . 206

**Editor's Letter-Box.**—Gorlaston Cottage Hospital . . . 211

**The Practitioner's Mirror and Retrospect:—**

Medicine—Hepatic Cirrhosis—Urticaria . . . 207

Surgery—Cataract, 209; Noises in the Ears—Treatment of Cold Abscess by Iodoform—Spontaneous Restoration of the Membrana Tympani—Removal of Deformity of Rickety Limbs—A Wandering Liver, 210; Hydatid Cysts of Liver . . . 211

**Scraps and Gleanings** . . . 211

**The Student of Medicine**—Professor Humphry on the Human Form — Leading Athletes — Appointments — Students' Medical Societies—Notes from the Schools—The Students' Bookshelf—Pass Lists—Athletics . . . 212

**General Charities.**

EXTRA SUPPLEMENT—THE NURSING MIRROR.

**En Passant.**—A Merry Christmas—Short Items—A Manchester Meeting—Queen Victoria Nurses—Those Pro's. Again . . . lxv

**A Nurse's Christmas-Box—£10,000** . . . lxvi

**Everybody's Opinion.**—An Appeal—Male Nurses—A Wedding Gift—Ladies' Committees . . . lxvii

**Death in Our Ranks** . . . lxvii

**Notes and Queries** . . . lxvii

**Christmas at Vancouver** . . . lxviii

**Presentation** . . . lxviii

**A Christmas Legend** . . . lxviii

**For Reading to the Sick.**—Christ in our Hearts . . . lxix

**Off Duty.**—A Winter Holiday (*continued*) . . . lxix

**New Competition—Amusements and Relaxation** . . . lxx

